# Age-dependent effects of chronic traumatic and social isolation stress on mice social behavior

**DOI:** 10.1016/j.ynstr.2025.100773

**Published:** 2025-11-27

**Authors:** Moonseok Choi, Jisu Jeong, Dongsoo Kim, Hong Seok Choi, Junghwa Ryu, Hye Jin Choi, Mookyung Cheon, Yun Ha Jeong

**Affiliations:** a Department of Neurodegenerative Diseases Research Group, Korea Brain Research Institute (KBRI), 61, Cheomdan-ro, Dong-gu, Daegu, 41062, Republic of Korea; b Department of Dementia Research Group, Korea Brain Research Institute (KBRI), 61, Cheomdan-ro, Dong-gu, Daegu, 41062, Republic of Korea; c Graduate School, Daegu Gyeongbuk Institute of Science and Technology (DGIST), Daegu, 42988, Republic of Korea

**Keywords:** Chronic traumatic stress, Chronic isolation stress, Adolescence, Adulthood, Life cycle, Social dominance behavior

## Abstract

Modern people are exposed to various stressful situations. Stress is a significant factor in emotional changes and social behavior and is associated with imbalances in physiological and psychological homeostasis, including brain function and structure. Stress has multiple causes, each of which has different impacts on social behavior throughout life. However, little is known about how stress influences social behavior across the life cycle. To understand this further, this study exposed mice to chronic stress at three different ages: adolescence, early adulthood, and adulthood. Chronic stresses were induced by combining chronic traumatic and isolation stress. Chronic stress has been shown to enhance social dominance behavior, especially in adolescence, and changes in the expression of genes and proteins, including Fabp7 and Cxcl12, were observed to change in the opposite direction compared to adulthood, confirming that it is related to changes in social dominance behavior. This study may provide important insights into factors related to adolescence social behavior abnormalities.

## Highlights

• Adolescence stress increased social dominance, adulthood stress reduced it.
• Dual chronic stressor model revealed age-dependent behavioral and gene changes.
• Hippocampal transcriptome showed distinct adolescence vs adulthood modules.
• *Fabp7* upregulated and *Cxcl12* downregulated in adolescence stress-exposed mice.
• Findings highlight Fabp7 and Cxcl12 as regulators of stress-induced behavior.

## 1 Introduction

Stress is widely known as a mental, physical, emotional, and behavioral response that occurs when individuals are exposed to difficult situations (Cohen et al., 2007). In addition, long-term exposure to stressful situations can have a significant impact on brain function and cause physiological, psychological, and behavioral changes (James et al., 2023; Yaribeygi et al., 2017). Childhood is a critical period for brain development, during which individuals rapidly acquire cognitive, emotional, and social skills through positive interactions with the people around them (Tierney and Nelson, 2009). People who repeatedly experience extreme stress in childhood, such as child abuse or separation from their parents, are more likely to have physical or mental disorders than those who experience stress in adulthood (Schneiderman et al., 2005).

Posttraumatic stress disorder (PTSD) is a stress-related cognitive and emotional disorder that occurs after exposure to stressful or traumatic events, such as war, torture, natural disaster, or frightening events (Adami et al., 2006; Yehuda, 2002). Neuroimaging studies have demonstrated PTSD-induced structural and functional abnormalities in the hippocampus (Liberzon and Martis, 2006). Single-prolonged stress (SPS) is a widely used model of PTSD in which mice are subjected to physical restraint, forced swimming, or ether anesthesia (Liberzon et al., 1997; Nahvi et al., 2019). Rats exposed to this SPS exhibit increased negative feedback in the HPA axis and decreased plasma corticosterone levels, resulting in a phenotype similar to the neuroendocrine features of human PTSD (Liberzon et al., 1999). Chronic social isolation (SI) stress contributes significantly to a wide range of mental health vulnerabilities, including loneliness, helplessness, depression, anxiety, and suicide (Brandt et al., 2022).

Studies have shown that chronic stress can change social dominance behavior (D'Amato et al., 2001; Williamson et al., 2017). Dominant interactions between individuals create a social structure in which hierarchy is determined by the relative dominance status of group members (Koski et al., 2015). Dominance status is essential for social stability and the survival and health of individual animals (Zhou et al., 2018). Several brain regions, including the amygdala, hippocampus, hypothalamus, striatum, intraparietal sulcus, ventromedial prefrontal cortex, and dorsolateral prefrontal cortex are known to be involved in the perception and learning of social dominance (Singer, 2012). Recently, hippocampal neurogenesis dysfunction has been linked to social dominance behavior (Grieco et al., 2025) and neural activity in the hypothalamus has been implicated in modulating both aggressive and defensive behaviors (Falkner et al., 2014; Wang et al., 2015), highlighting the pivotal role of the hippocampus and hypothalamus in regulating social dominance.

Stress and social behavior are deeply entangled (Beery and Kaufer, 2015). However, little is known about the impact of stress on social behavior across life cycles. We hypothesized that stress exposure at each developmental stage would differentially affect social behavior and its molecular correlates. To investigate this hypothesis, we subjected mice to chronic stresses at three different ages: adolescence, early adulthood, and adulthood. We established chronic stress paradigms by combining chronic traumatic and social isolation stresses. We found that chronic stress specifically enhanced social dominance behaviors during adolescence and that stress-induced changes in *Fabp7* and *Cxcl12* gene expression were associated with social dominance behaviors. Our results suggest that changes in social behavior and gene expression that are unique to adolescence may provide clues for further research into adolescence social behavior abnormalities.

## 2 Materials and methods

### *2.1* Animals

C57BL/6J male mice were obtained from Jackson Laboratories (JAX: #00064, USA) and maintained. The mice were housed in groups of three to five mice per cage with ad libitum access to food and water under a 12-h light‒dark cycle (lights on at 08:00 a.m.), a humidity of 55 ± 5 %, and a temperature of 25 ± 1 °C. The experimental protocols conducted in this study were ethically reviewed and approved by the Institutional Animal Care and Use Committee (authorization approval number: IACUC-23-00042) of the Korea Brain Research Institute (KBRI).

### *2.2* Experimental design

To investigate the effects of stress according to age, we established three different age groups of C57BL/6J mice: 3 weeks (Adolescence), 16 weeks (Early adulthood), and 40 weeks (Adulthood). In each life stage, the mice were randomly divided into two groups: the Unstressed Group-housed (control, n = 10) and the chronic stress (stress, n = 10) groups during adolescence; the control (n = 7) and stress (n = 8) groups during early adulthood; and the control (n = 8) and stress (n = 9) groups during adulthood. Each group was exposed to chronic traumatic stress for 1 week following chronic SI for 5 weeks. After chronic stress, behavioral alterations were evaluated via the tube dominance test, the forced swimming test (FST), and the tail suspension test (TST). Blood was collected twice each after chronic traumatic and SI stresses. All the animals were sacrificed after the behavior tests.

#### *2.2.1* Chronic traumatic stress

To examine the effects of traumatic stress, we administered acute electrical stress (footshock) and Single-prolonged stress (SPS). Chronic traumatic stress was applied for seven consecutive days. For electrical stress, the mice were exposed to inescapable foot shock on the grid of a shuttle box (41.5 cm × 21 cm × 3.5 cm). Electric shocks were delivered randomly at 1–3 mA for 1–10 s, and this process was repeated seven times. After electrical stress, the mice were given a 15–30 min rest period. After rest, the mice were exposed to SPS. In the SPS procedure, the mice were subjected to restraint stress for 3 h and then allowed to swim for 10 min. After forced swimming stress, the mice were dried thoroughly and allowed to recover for approximately 15 min. Finally, the mice were exposed to isoflurane in the anesthesia chamber for 30 s to 1 min until they lost consciousness. All mice were subsequently returned to their home cages. The body weights of the mice were measured before and after the day of chronic traumatic stress.

#### *2.2.2* Chronic social isolation stress

To enhance the effects of chronic traumatic stress, mice were subjected to social isolation stress. One week after the chronic traumatic stress, the mice were housed alone for five weeks. The control mice were not stressed and were housed in groups of 3–5 mice during the experimental period.

### *2.3* Tube dominance test

The tests were performed with minor modifications according to the instructions (Fan et al., 2019). The mice were handled gently for 5 min daily for at least 1 week prior to the tube dominance experiments. The tube device was a transparent plexiglas tube measuring 30 cm in length and 3.5 cm in internal diameter, with a removable door in the center. Notably, age and weight can affect the results of the tube test. Therefore, we tested a group of similar ages and weights in each group. To reduce stress and anxiety, the mice were acclimated to the behavioral testing room for at least 1 h prior to the experiment. For training, all the mice were given 10 trials per day, with 5 trials from each side. On test days, two animals were positioned at opposite ends of the tube and then released. After a power struggle in the center of the tube, the mouse that pushes or moves its opponent to make him retreat first is declared the "winner." If the mouse cannot withstand the pressure and retreats out of the tube, it is declared a "loser". If all four legs of the mouse touch the outside surface of the tube, it is defeated. At the end of each trial, the mice returned to their home cages for at least 1 min before starting the next trial to minimize the influence of recent wins or losses. To obtain reliable data, each mouse was tested on three consecutive days to determine winners and losers. The winner receives 1 point, the loser −1 point. In case of a tie, 0 points are awarded. Using a binary system, wins were calculated as a percentage of points scored in the entire match.

### *2.4* Forced swimming test

A transparent, cylindrical acrylic tank (height: 25 cm, diameter: 10 cm) was prepared and filled approximately two-thirds with water (23–25 °C). The water depth was adjusted to prevent the mice tails and feet from touching the bottom, and to prevent escape but not drowning. After acclimating the mice to the laboratory for 30 min prior to the experiment, each mouse was placed in the tank simultaneously and the experiment began. The test lasted 6 min and was videotaped. The last 4 min were analyzed, excluding the first 2 min for adaptation purposes. Immobility was defined as the absence of active movement for escape, and the minimum movement necessary to keep the mice head above water to avoid drowning was considered immobility. Immobility time (in seconds) was quantified from video recordings with blinded manual scoring.

### 2.5 Tail suspension test

Mice were acclimated to the testing room for 30 min before the experiment. A 30-cm-high suspension bar was installed above the experimental bench, and partitions were installed to prevent visual contact between the mice. The experiment was videotaped. Each mouse was secured to the horizontal bar with medical adhesive tape 2–3 mm from the tip of its tail. The experimental time was recorded for a total of 6 min, excluding the initial 2 min acclimation period, and the last 4 min were analyzed. Immobility was defined as passive suspension without any proactive movement or escape attempts. Small forelimb tremors without hindlimb involvement or inertial pendulum movements were not considered activity. Total immobility time was quantified from video recordings using blinded manual scoring.

### *2.6* Blood collection and corticosterone enzyme-linked immunosorbent assay

Blood was collected via retro-orbital sinus/plexus sampling under isoflurane anesthesia 30 min after the last behavioral analysis. After the blood was collected, it was left at room temperature to clot. The blood was centrifuged at 3000 rpm at 4 °C for 10 min to separate clots and serum. All serum samples were collected and stored at −80 °C until analysis. Serum corticosterone (CORT) concentrations were measured via a CORT competitive enzyme-linked immunosorbent assay (ELISA) kit (Arbor Assays, Cat. #K014-H5; Ann Arbor, MI, USA) according to the manufacturer's protocol. The optical density at 450 nm was read on a Spectra Max iD5 microplate reader (San Jose, CA, USA). The absorbance values were converted into concentrations using four parameters, and the serum CORT concentration was quantified in triplicate by comparison with the standards.

### *2.7* Brain harvest

After blood extraction, anesthesia was induced via intraperitoneal injection of 2,2,2-tribromoethanol (150 mg/kg, Avertin, Sigma, St. Louis, MO, USA). The mice were euthanized through perfusion with 1 × phosphate-buffered saline (PBS, pH 7.2) for 5 min. To perform an immunofluorescence experiment, brain hemispheres were initially fixed in 4 % paraformaldehyde for 24 h. The other half of the brain were stored at −20 °C for RNA experiments.

### *2.8* RNA-sequencing analysis

#### *2.8.1* RNA isolation

For RNA extraction, we used an RNeasy Plus Micro Kit from Qiagen (RNeasy Plus Micro Kit #74034, Hilden, Germany). Total RNA was isolated from the hippocampus and hypothalamus, and the purity and quantity of RNA were measured via a NanoDrop system. Furthermore, RNA integrity was further analyzed via the Agilent 4200 Tape Station.

#### *2.8.2* RNA-seq data analysis

Quality control of the raw sequencing data was conducted with FastQC v.0.12.1 (Andrews, 2010). High-quality reads were retained and aligned to the mouse reference genome (GRCm38) via STAR v.2.0.5 (Dobin et al., 2013). Gene-level quantification was performed through HTSeq-count v.2.0.5 (Anders et al., 2015).

Analysis of differentially expressed genes (DEGs) was performed using the EdgeR R package (Robinson et al., 2010). Gene counts were normalized using the trimmed mean of M-values (TMM) method to account for compositional differences between the libraries. The normalized counts were fitted to a quasilikelihood (QL) generalization of the negative binomial (NB) model. The DEGs were assessed via QL F tests, which involved constructing a design matrix and selecting coefficients to be tested under the NB GLM framework. Based on these test results, the DEGs were defined with a *p*-value threshold of 0.05 and an absolute log_2_-fold change greater than 0.4. Using these criteria, we identified the DEGs in the following three comparisons: (1) adolescence: stress vs control, (2) adulthood: stress vs control, and (3) stress: adulthood vs adolescence.

#### *2.8.3* Gene set enrichment analysis

Gene Ontology (GO) and Kyoto Encyclopedia of Genes and Genomes (KEGG) pathway enrichment analyses were performed via the gprofiler2 R package (Kolberg et al., 2020) to functionally interpret the DEGs. Among the enriched results, GO biological process (BP) and KEGG pathway terms related to neural function were selected as the main functional annotations. These selected terms were visualized through the circlize R package (Gu et al., 2014) as chord diagrams, highlighting the relationships between genes and their associated functional categories.

### *2.9* Quantitative PCR analysis

Quantitative PCR (qPCR) was used to analyze the mRNA expression levels of candidate genes on a QuantStudio 5 Fast Real-Time PCR system (Applied Biosystems, Monza, Italy) with Fast SYBR Green Master Mix (Applied Biosystems, Monza, Italy). The PCR cycling conditions were as follows: 1 s at 96 °C (denaturing), 40 cycles of 20 s at 60 °C (annealing/extension) in the PCR stage, 1 s at 96 °C, 20 s at 60 °C, and 1 s at 95 °C in the melting curve stage. The results were normalized to the relative expression level of mRNA for the target genes and analyzed via the comparative CT method, with GAPDH as the reference gene internal control. NCBI Primer-BLAST was used for primer design for the candidate genes. The analysis of the melting curves was used to determine the specificity of the PCR products.

### *2.10* Immunofluorescence staining

Fixed brain hemispheres were immersed in a 30 % sucrose solution for 48 h to facilitate cryoprotection. Afterwards, brain molds were created via optimal cutting temperature (OCT) compound and sectioned into 30 μm-thick coronal sections via a cryostat. (Cryostat #CM1860, Leica Biosystems, Newcastle, UK). All sectioned brain slices were stored at −20 °C in cryoprotection buffer prior to biochemical analysis. The immunofluorescence staining procedures followed previously established methods (Choi et al., 2023). The brain sections were washed three times with PBS and then permeabilized with ice-cold methanol at −20 °C for 10 min. Antigen retrieval was performed by immersing the sections in a 10 mM citric acid (pH 6.0) solution, heating them in a 95 °C water bath for 30 min, and washing them three more times with PBS. The sections were then blocked with a solution containing 2 % bovine serum albumin and 0.3 % Triton X-100 in PBS for 1 h. Primary antibodies against Fabp7 (mouse, 1:1000, NBP3-05531, Novus Biologicals, Littleton, CO, USA), Egr2 (rabbit, 1:2000, ab245228, Abcam, Cambridge, UK), and Cxcl12 (mouse, 1:100, MA5-23759, Invitrogen, Waltham, MA, USA) were applied overnight at 4 °C. Subsequently, the secondary antibodies anti-rabbit-488 (Donkey, 1:200, A21206, Thermo Fisher, Waltham, MA, USA), anti-rabbit-555 (Donkey, 1:200, A31572, Thermo Fisher, Waltham, MA, USA), anti-mouse-488 (Donkey, 1:200, A21202, Thermo Fisher, Waltham, MA, USA) and anti-mouse-555 (Donkey, 1:200, A31570, Thermo Fisher, Waltham, MA, USA) were applied for 2 h at 24 °C. Finally, the brain sections were counterstained with To-pro (1:1000, T3605, Thermo Fisher, Waltham, MA, USA) or 4′,6-diamidino-2-phenylindole (DAPI) for nuclear visualization and mounted for observation. Confocal microscopy (Confocal-A1R-MP, Nikon Corporation, Tokyo, Japan) was utilized for imaging. For quantitative image analysis, confocal maximum projection images were processed using ImageJ software (NIH, Bethesda, MD, USA). Each fluorescence channel was separated and converted to 8-bit images, and regions of interest (ROIs) were defined in the hippocampus (dentate gyrus) and hypothalamus (arcuate nucleus). The fluorescence intensity and area within the ROIs were then measured to quantify protein expression levels. Three to five tissue sections per animal were averaged and analyzed.

### *2.11* Statistical analysis

The data are presented as the mean ± standard error of the mean (SEM). Statistical analyses were conducted via PRISM 10 software (GraphPad, La Jolla, CA, USA), employing an unpaired *t*-test or one-way or two-way analysis of variance (ANOVA) followed by Tukey's honestly significant difference (HSD) post hoc test to determine statistical significance. The significance level of *p* < 0.05 was considered statistically significant.

All experiments were conducted in compliance with the ARRIVE (Animal Research: Reporting of In Vivo Experiments) guidelines.

## 3 Results

### 3.1 Chronic stress in adolescence reinforces social dominance behavior

**Fig. 1:**
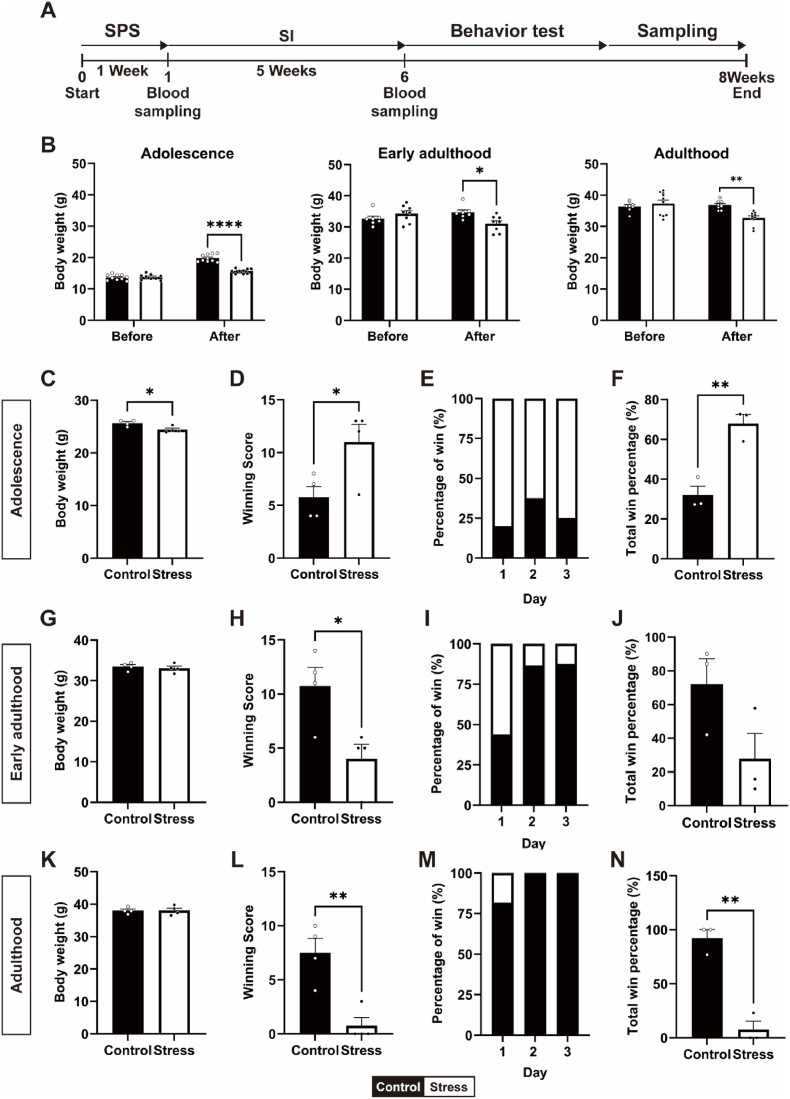
Effects of chronic stress on social dominance behavior in life cycles. (A) Overall experimental scheme (B) Effects of chronic traumatic stress on body weight changes (Two-way ANOVA with Tukey's multiple comparison test; adolescence, n = 9–10 mice per group, ∗∗∗∗*p* < 0.0001; early adulthood, n = 7–8 mice per group ∗*p* < 0.05; adulthood, n = 8–10 mice per group, ∗∗*p* < 0.01). Dominant and subordinate behavior of the control and stress groups in the tube dominance test: (C, G, K) body weight (Student's t-test, ∗*p* < 0.05, n = 4 mice per group), (D, H, L) winning score (Student's t-test, ∗*p* < 0.05, ∗∗*p* < 0.01, n = 4 mice per group), (E, I, M) percentage of win, and (F, J, N) total win percentage (Student's t-test, ∗∗*p* < 0.01, n = 4 mice per group). Results are presented as the mean ± SEM. Statistical analysis was performed using Two-way ANOVA or Student's t-test (unpaired). ∗*p* < 0.05, ∗∗*p* < 0.01 ∗∗∗∗*p* < 0.0001. ns: no significance.

A schematic diagram of experimental procedures was shown in Fig. 1A. We monitored body weight changes throughout the week of chronic traumatic stress. Compared with those of the control group, the body weights of the stress group decreased for 1 week of chronic traumatic stress at all age groups. In adolescence, two-way ANOVA revealed a statistically significant interaction between the control and stress groups. (F_1, 34_ = 49.71, ∗∗∗∗*p* < 0.0001). In early adulthood, the stressed group had lower body weights than did the control group (F_1, 26_ = 8.414; ∗*p* < 0.05). Similarly, in adulthood, the body weight of the stressed group tended to decrease compared with that of the control group (F_1, 31_ = 8.711, ∗∗*p* < 0.01). No difference was observed in the body weight of each group before stress (*p* = 0.9873 in adolescence; *p* = 0.5955 in early adulthood; *p =* 0.8926 in adulthood) (Fig. 1B).

To determine whether chronic stress affect social dominance behavior in mice, a tube dominance test was performed via a well-established behavioral paradigm (Fan et al., 2019). The age and weight of the mice potentially influenced the results of the tube test. Therefore, similar ages and weights of the mice in each age group (within a 15 % difference) were used (adolescence; t_(6)_ = 3.034, ∗*p* < 0.05, early adulthood; t_(6)_ = 0.6158, *p* = 0.5606, adulthood; t_(6)_ = 0.05512, *p* = 0.9578) (Fig. 1C–G, K). Owing to the weight-matching paradigm, not all the mice in each group could be used for the tube test. Thus, we used 4 mice in each group across all age groups. In the training sessions, the mice were considered habituated if they traversed both sides of the tube without hesitation. During the test trial, the mouse pushing another mouse out of the tube was designated a 'winner', and the mouse with all four paws retreated from the tube as a 'loser'. After habituation and training, the control and stress groups of mice were matched 16 times per day for three days, and their positions were switched daily. To prevent mouse fatigue from repeated matches, we designed our test matches in a random order so that the same mouse was never used in consecutive games for at least 1 min. Interestingly, the differences in the results of the tube dominance test between control and stressed mice were reversed in adolescence compared with early adulthood and adulthood. (Fig. 1D–H, L). The stress group had significantly higher winning scores than did the control group during adolescence (t_(6)_ = 2.660, ∗*p* < 0.05) (Fig. 1D). However, the stress group had lower winning scores than did the control group in early adulthood (t_(6)_ = 3.104, ∗*p* < 0.05) and adulthood (t_(6)_ = 4.439, ∗∗*p* < 0.01) (Fig. 1H–L). Winning scores were calculated by averaging the number of wins for each mouse (Fig. 1D–H, L).

We found that dominant or subordinate behaviors in the stress groups were reinforced over consecutive trial days. In adolescence, the stress group consistently presented a greater percentage of win grades (Day 1: 80, Day 2: 62.5, Day 3: 75(%)) than did the control group (Day 1: 20, Day 2: 37.5, Day 3: 25(%)) (Fig. 1E). On the other hand, in early adulthood and adulthood, the control group consistently presented higher win scores (Early adulthood, Day 1: 43.8, Day 2: 86.7, Day 3: 87.5(%); Adulthood, Day 1: 81.82, Day 2: 100, Day 3: 100(%)) than did the stress group (Early adulthood, Day 1: 56.3, Day 2: 13.3, Day 3: 12.5(%); Adulthood, Day 1: 18.18, Day 2: 0, Day 3: 0(%)) (Fig. 1I–M). Therefore, the total percentage of winners in the stress group was significantly greater than that in the control group in the adolescence stage, suggesting that chronic stress increased social dominance behavior during adolescence (t_(4)_ = 5.659, ∗∗*p* < 0.01) (Fig. 1F). However, the stress group in adulthood presented significantly lower total win percentages than did the control group, unlike adolescence (t_(4)_ = 7.7779, ∗∗*p* < 0.01) (Fig. 1N). The above results showed that the same stress paradigm had different effects on social dominance depending on age.

To examine the effects of stress on stress hormones, we measured serum CORT levels after chronic stress across three developmental age stages: adolescence, early adulthood, and adulthood. According to ELISA-based quantification, serum CORT levels were significantly greater in all chronic traumatic stress exposure groups than in the control groups across all developmental stages (Supplementary Fig. 1A) (adolescence: t_(17)_ = 5.479, ∗∗∗∗*p* < 0.0001; early adulthood: t_(13)_ = 3.979, ∗∗*p* < 0.01; adulthood: t_(24)_ = 7.251, ∗∗∗∗*p* < 0.0001). However, the levels of CORT decreased after chronic SI stress in the stress group compared with those in the control group during adolescence and adulthood. (adolescence: t_(8)_ = 2.898, ∗*p* < 0.05; adulthood: t_(24)_ = 2.406, ∗*p* < 0.05). In early adulthood, the difference was not significant. (t_(13)_ = 1.849, *p* = 0.0874) (Supplementary Fig. 1B). These results suggest that the regulation of the endocrine stress response varies with stress type: chronic traumatic stress vs chronic social isolation stress.

To determine whether chronic stress affect depression-like behavior, FST and TST were performed. During the FST, immobility time did not differ significantly between the control and stress groups at any age (Supplementary Fig. 2A). This may be attributed to exposure to swimming stress during chronic traumatic stress, especially in the stress group. However, during the TST, a significant increase in immobility time was observed in both the adolescence and adulthood stress groups (Adolescence: t_(17)_ = 2.522, ∗*p* < 0.05; Adulthood: t_(9)_ = 2.306, ∗*p* < 0.05), suggesting that chronic stress induced depressive-like behaviors, although this effect was not seen in early adulthood (Supplementary Fig. 2B). Taken together, these results suggest that both hormonal and behavioral endpoints effectively reflect stress-induced changes, demonstrating the reliability of the stress model across the life cycles.

### *3.2* Identified upregulated or downregulated genes in adolescence after chronic stress

**Fig. 2:**
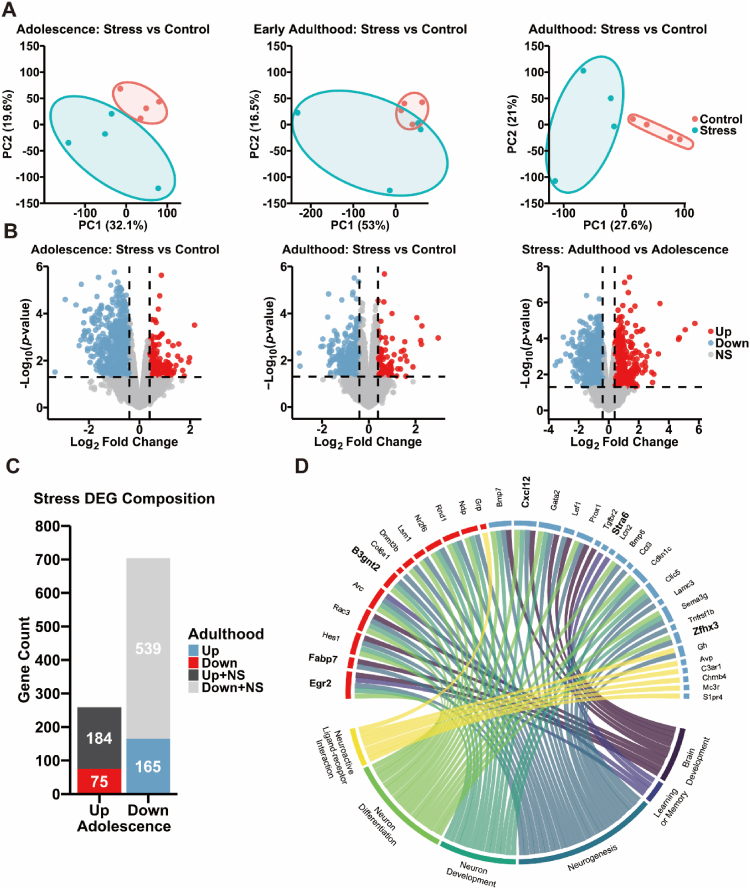
RNA-seq analysis of stress-induced gene expression changes across li**fe cycles.** (A) PCA plot of hippocampal transcriptomes (Adolescence, Stress n = 4, Control n = 4; Early adulthood, Stress n = 4, Control n = 4; Adulthood, Stress n = 4, Control n = 4). Stress and control groups were clearly separated in adolescence and adulthood, but not in early adulthood. (B) Volcano plot showing DEGs (*p* < 0.05 and |log_2_FC| > 0.4) in comparisons of adolescence: stress vs control, adulthood: stress vs control and stress: adulthood vs adolescence groups. The x-axis indicates log_2_FC and the y-axis represents –log_10_ (p-value). (C) Stress DEG composition based on opposite expression pattern. Among 259 up-regulated genes in adolescence: stress vs control (left), 75 overlapping genes (red) that were down-regulated in stress: adulthood vs adolescence; 184 non-significant or up-regulated genes (black). Among 704 down-regulated genes in adolescence: stress vs control (right), 165 overlapping genes (sky blue) that were up-regulated in stress: adulthood vs adolescence; 539 non-significant or down-regulated genes (gray). (D) Among 240 (75 + 165) stress response genes, 35 enriched genes were identified using enriched GO terms related to neural function and KEGG pathway.

To determine whether chronic stress influence gene expression changes along with changes in social dominance behavior, we performed transcriptome analyses of the hippocampus, a brain region involved in social dominance, at three different age stages. Principal component analysis (PCA) was performed on all samples according to age and stress conditions. In both adolescence and adulthood groups, the stress and control samples presented marked separation, indicating that stress-induced transcriptomic changes were evident in these stages. In contrast, early adulthood samples showed no significant difference between the stress group and the control group (Fig. 2A). Based on these results, subsequent analyses focused on adolescence and adulthood groups.

Significantly differentially expressed genes (DEGs) were identified with a threshold of *p* < 0.05 and absolute log_2_-fold change (|log_2_FC|) > 0.4 across all three comparisons: (1) adolescence: stress vs control, (2) adulthood: stress vs control, and (3) stress: adulthood vs adolescence. This analysis revealed 259 upregulated genes and 704 downregulated genes in the adolescence (stress vs control) comparison, 110 upregulated genes and 443 downregulated genes in the adulthood (stress vs control) comparison, and 733 upregulated genes and 1197 downregulated genes in the stress (adulthood vs adolescence) comparison (Fig. 2B).

To further analyze the changes in DEGs according to stress, we compared and analyzed the changes in upregulated and downregulated DEG expression patterns between adolescence and adulthood. Among the 259 (184 + 75) genes whose expression was upregulated after stress in the adolescence stress group, 75 genes were downregulated in the adulthood stress group. Conversely, among the 704 (539 + 165) genes downregulated after stress in the adolescence stress group, 165 genes were upregulated in the adulthood stress group (Fig. 2C). To assess the biological relevance of the opposing transcriptional patterns of gene expression changes in the adolescence and adulthood groups after stress, we performed GO enrichment analysis. The 240 (75 + 165) stress response genes that were expressed oppositely in adolescence and adulthood were analyzed via enriched GO terms related to neural function, resulting in 35 enriched genes (Fig. 2D). Specifically, 13 genes (red) were derived from upregulated genes in adolescence, and 22 genes (sky blue) were downregulated genes in adolescence. The enriched GO terms associated with neural function included neuron differentiation (19 genes), neurogenesis (26 genes), neuron development (14 genes), brain development (10 genes), and learning or memory (4 genes). Additionally, neuroactive ligand‒receptor interactions (7 genes) were identified through KEGG pathway analysis (Table 1). For the 35 genes used in the enrichment analysis, we examined their *p*-value and log_2_FC changes in adolescence: stress vs control comparison (Supplementary Table 1).

**Table 1:** Classification of 35 enriched genes among upregulated and downregulated stress response genes in the adolescence stress group using GO term analysis related to neural function.

DEG pathway	Total gene numbers	Upregulated	Downregulated
Neuron differentiation	19	*Arc, B3gnt2, Dnmt3b, Egr2, Hes1, Lsm1, Nr2f6, Rac3, Rnd1,Ndp*	*Bmp6, Bmp7, Cdkn1c, Clic5, Cxcl12, Gata2, Prox1, Sema3g, Zfhx3*
Neurogenesis	26	*Arc, B3gnt2, Col6a1, Dnmt3b, Egr2, Fabp7, Hes1, Lsm1, Nr2f6, Rac3, Rnd1, Ndp*	*Bmp6, Bmp7, Ccl3, Cdkn1c, Clic5, Cxcl12, Gata2, Lamc3, Lef1, Prox1, Sema3g, Tnfrsf1b, Zfhx3, Gh*
Neuron development	14	*Arc, B3gnt2, Egr2, Hes1, Nr2f6, Rac3, Rnd1, Ndp*	*Bmp7, Cdkn1c, Clic5, Cxcl12, Gata2, Sema3g*
Learning or memory	4	*Arc, Egr2*	*Stra6, Len2*
Brain development	10	*Egr2, Fabp7, Hes1, Rac3*	*Bmp7, Cxcl12, Gata2, Lef1, Prox1, Tgfbr2*
Neuroactive ligand-receptor interaction	7	*Grp*	*Avp, C3ar1, Chrnb4, Mc3r, S1*pr*4, Gh*

### *3.3* Validation of the expression levels of 35 selected genes in the hippocampus and hypothalamus

**Fig. 3:**
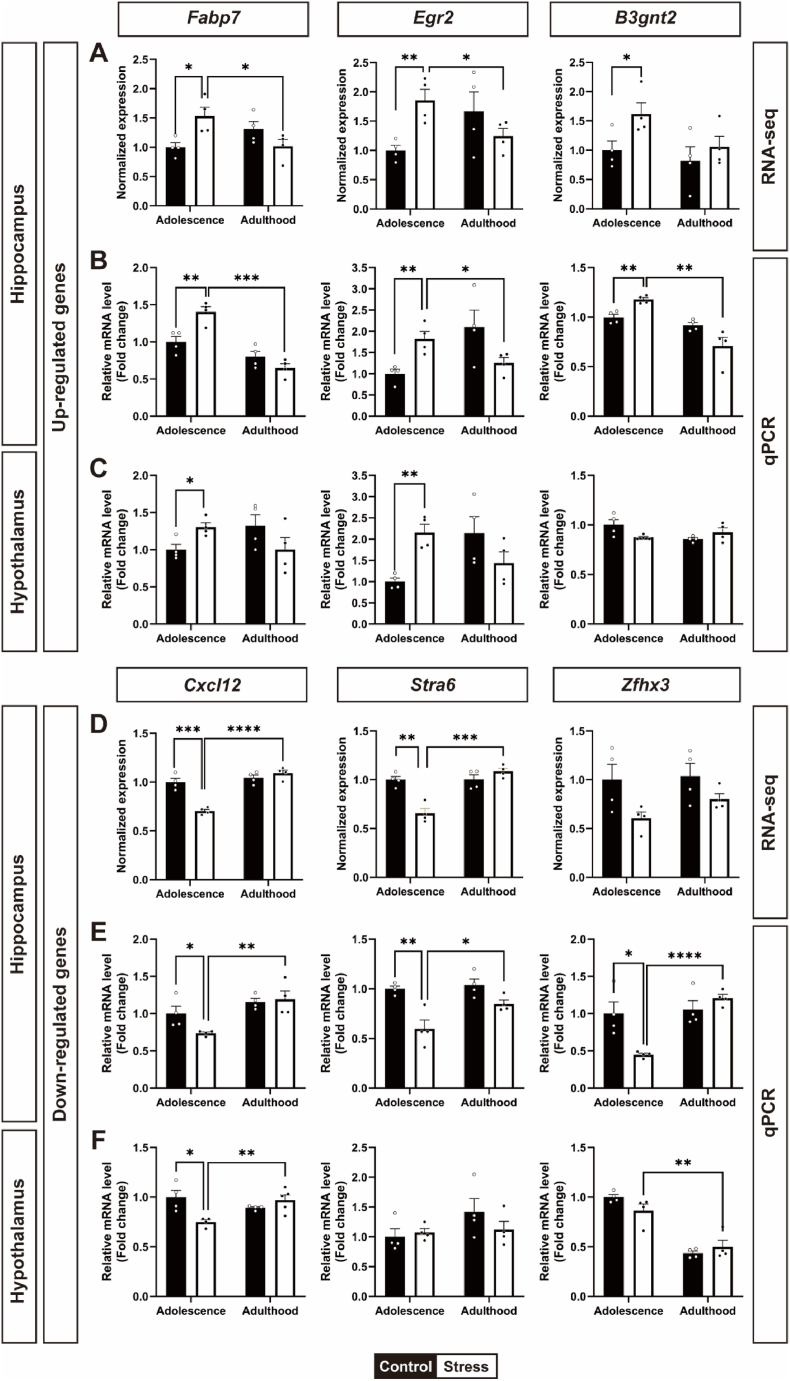
RT-qPCR validation of selected differentially expressed genes in the hippocampus and hyp**othalamus.** (A) Upregulated genes from RNA-seq data of the hippocampus. (B, C) Validation of RNA-seq data using qPCR in the hippocampus and hypothalamus. (D) Downregulated genes from RNA-seq data of the hippocampus. (E, F) Validation of RNA-seq data using qPCR in the hippocampus and hypothalamus. All data are presented as mean ± SEM. ∗*p* < 0.05, ∗∗*p* < 0.01, ∗∗∗*p* < 0.001, ∗∗∗∗*p* < 0.0001 the control and stress groups in adolescence or adolescence vs adulthood in the stress group (n = 4 in each group, Student's t-test).

After GO terms related to neural function were analyzed, qPCR was performed to validate the mRNA expression levels of 35 selected genes, and the RNA-seq and mRNA expression patterns were confirmed to be consistent for 6 genes: the upregulated genes *Fabp7, Egr2*, and *B3gnt2* and the downregulated genes *Cxcl12*, *Stra6*, and *Zfhx3* (Fig. 3). The target genes were first normalized to *Gapdh*, and the relative fold changes in mRNA expression were compared to that of the adolescence control group, which was set to 1.

According to the RNA-seq analysis, stress exposure during adolescence increased the expression of *Fabp7* (t_(6)_ = 3.083, ∗*p* < 0.05), *Egr2* (t_(6)_ = 4.111, ∗∗*p* < 0.05) and *B3gnt2* (t_(6)_ = 2.524, ∗*p* < 0.05) in the stress group compared with the control group. Moreover, the expression of these genes in the stress group during adolescence was also significantly greater than that in the stress group during adulthood *Fabp7* (t_(6)_ = 2.695 ∗*p* < 0.05), *Egr2* (t_(6)_ = 2.646, ∗*p* < 0.05), and *B3gnt2* (t_(6)_ = 2.134, *p* > 0.05) (Fig. 3A). The gene expression patterns of these three genes identified via RNA-seq were identical to those identified via qPCR analysis in the hippocampus. The relative mRNA expression levels of these three genes were significantly greater in the stress group than in the control group during adolescence [*Fabp7* (t_(6)_ = 3.881, ∗∗*p* < 0.01), *Egr2* (t_(6)_ = 4.027, ∗∗*p* < 0.01), and *B3gnt2* (t_(6)_ = 4.968, ∗∗*p* < 0.01)]. Similar to the RNA-seq data, the relative mRNA expression levels of *Fabp7*, *Egr2*, and *B3gnt2* in the stress group during adolescence were markedly greater than those in the stress group during adulthood [*Fabp7* (t_(6)_ = 8.035, ∗∗∗*p* < 0.001), *Egr2* (t_(6)_ = 2.621, ∗*p* < 0.05), and *B3gnt2* (t_(6)_ = 5.040, ∗∗*p* < 0.01)] (Fig. 3B). We also examined the expression patterns of these genes in the hypothalamus, a brain region involved in social dominance. Interestingly, similar expression patterns were observed between the hippocampus and hypothalamus in *Fabp7* (t_(6)_ = 3.227, ∗*p* < 0.05, stress vs control in adolescence; t_(6)_ = 1.732, *p* = 0.1340, stress in adolescence vs stress in adulthood). However, the *Egr2* mRNA expression level in the hypothalamus was significantly greater only in the adolescence stress group than in the control group (t(6) = 5.565, ∗∗*p* < 0.01). However, no significant differences in the mRNA expression levels of *B3gnt2* in the hypothalamus were detected between the groups (Fig. 3C).

We also found downregulated genes after stress exposure in the stress group of adolescence compared with the control group of adolescence and stress groups of adulthood through RNA-seq analysis: *Cxcl12* (t_(6)_ = 7.026, ∗∗∗*p* < 0.001, stress vs control in adolescence; t_(6)_ = 11.08, ∗∗∗∗*p* < 0.0001, stress in adolescence vs adulthood); *Stra6* (t_(6)_ = 5.806, ∗∗*p* < 0.01, stress vs control in adolescence; t_(6)_ = 7.568, ∗∗∗*p* < 0.001, stress in adolescence vs adulthood); and *Zfhx3* (t_(6)_ = 2.306, *p* > 0.05, stress vs control in adolescence; t_(6)_ = 2.271, *p* > 0.05, stress in adolescence vs adulthood) (Fig. 3D). Similarly, the gene expression patterns of these three downregulated genes identified via RNA-seq were similar to those identified via qPCR analysis of the hippocampus. The relative hippocampal mRNA expression levels of three genes (*Cxcl12*, *Stra6* and *Zfhx3*) in the adolescence in the stress group were much lower than those in the control group [*Cxcl12* (t_(6)_ = 2.584, ∗*p* < 0.05), *Stra6* (t_(6)_ = 4.314, ∗∗*p* < 0.01), and *Zfhx3* (t_(6)_ = 3.557, ∗*p* < 0.05)] and the stress groups of adulthood [*Cxcl12* (t_(6)_ = 4.022, ∗∗*p* < 0.01), *Stra6* (t_(6)_ = 2.696, ∗*p* < 0.05), and *Zfhx3* (t_(6)_ = 15.38, ∗∗∗∗*p* < 0.0001)] (Fig. 3E). In the hypothalamus, the relative mRNA expression level of the *Cxcl12* gene was similar to that in the hippocampus (t_(6)_ = 3.377, ∗*p* < 0.05, control vs stress in adolescence; t_(6)_ = 5.506, ∗∗*p* < 0.01, stress in adolescence vs adulthood), whereas the other genes did not (Fig. 3F). Therefore, these results confirmed that the expression patterns of the 6 genes validated by qPCR were consistent with the RNA-seq data in the hippocampus. The primer sequences are listed in the Supplementary Table 2.

### *3.4* Chronic stress-induced changes in Fabp7 and Cxcl12 protein expressions during adolescence

**Fig. 4:**
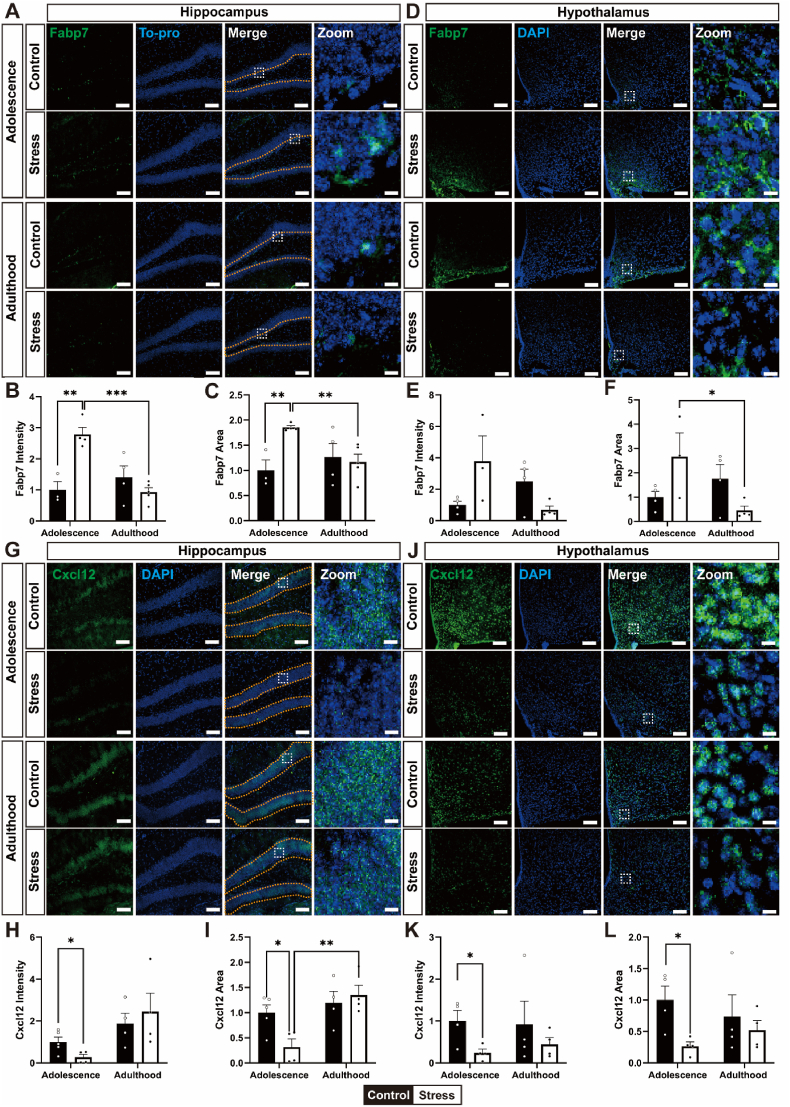
Effects of chronic stress on the protein expression levels of Fabp7 and Cxcl12 in the hippocampus and hypothalamus. (A) Representative confocal images showing the immunofluorescence staining of Fabp7 (green) in the hippocampus of the control and stress groups. Nuclei were counterstained with DAPI (blue). Scale bar: 100 μm, Zoom: 10 μm. (B, C) Quantification of the intensity and area of Fabp7 immunofluorescence in the hippocampus of the control and stress mice. (D) Representative confocal images showing the immunofluorescence staining of Fabp7 (green) in the hypothalamus of the control and stress groups. Scale bar: 100 μm, Zoom: 10 μm. (E, F) Quantification of the intensity and area of Fabp7 immunofluorescence in the hypothalamus of the control and stress groups. (G) Representative confocal images showing the immunofluorescence staining of Cxcl12 (green) in the hippocampus of the control and stress groups. Scale bar: 100 μm, Zoom: 10 μm. (H, I) Quantification of the intensity and area of Cxcl12 immunofluorescence in the hippocampus of the control and stress groups. (J) Representative confocal images showing the immunofluorescence staining of Cxcl12 (green) in the hypothalamus of the control and stress groups. Scale bar: 100 μm, Zoom: 10 μm. (K, L) Quantification of the intensity and area of Cxcl12 immunofluorescence in the hypothalamus of the control and stress groups. All data are presented as mean ± SEM (n = 3–5 mice per group). ∗*p* < 0.05, ∗∗*p* < 0.01, ∗∗∗*p* < 0.001 stress vs control in adolescence or adolescence vs adulthood in the stress group (unpaired *t*-test).

Changes in the mRNA expression of 35 genes selected through RNA-seq analysis were confirmed in the hippocampus and hypothalamus via qPCR. Among the six genes whose mRNA expression was verified through qPCR, the protein expression of Fabp7, Cxcl12, and Egr2, which presented identical mRNA expression patterns in the hippocampus and hypothalamus, was confirmed through immunofluorescence staining (Fig. 4). In the hippocampus, the intensity and area of Fabp7 immunofluorescence were significantly greater in the stress group than in the control group at adolescence (Fig. 4B and C). Specifically, Fabp7 intensity was elevated (t_(5)_ = 5.147, ∗∗*p* < 0.01, stress vs control in adolescence; t_(7)_ = 7.270, ∗∗∗*p* < 0.001, adolescence vs adulthood in the stress group), and the Fabp7-positive area was expanded (t_(5)_ = 4.766, ∗∗*p* < 0.01, stress vs control in adolescence; t_(7)_ = 3.878, ∗∗*p* < 0.01, adolescence vs adulthood in stress). However, in the hypothalamus, Fabp7 intensity was not altered (t_(5)_ = 2.048, *p* = 0.0959, control vs stress in adolescence; t_(5)_ = 2.275, *p* = 0.0720, adolescence vs adulthood in stress), and the Fabp7-positive area apparently expanded (t_(5)_ = 1.938, *p* = 0.1103, stress vs control in adolescence; t_(5)_ = 2.644, ∗*p* < 0.05, adolescence vs adulthood in stress) (Fig. 4E and F). For Cxcl12, the intensity of immunofluorescence was lower in the hippocampus of adolescence in the stress group than in the control group (t_(7)_ = 2.510, ∗*p* < 0.05, stress vs control in adolescence; t_(6)_ = 2.413, *p* = 0.0524, adolescence vs adulthood in stress), and the Cxcl12-positive area was smaller in the stress group than in the control group (t_(7)_ = 3.032, ∗*p* < 0.05, stress vs control in adolescence; t_(7)_ = 4.072, ∗∗*p* < 0.01, adolescence vs adulthood in stress) (Fig. 4H and I). Similarly, in the hypothalamus, Cxcl12-positive intensity (t_(6)_ = 2.861, ∗*p* < 0.05, stress vs control in adolescence; t_(6)_ = 1.133, *p* = 0.3006, adolescence vs adulthood in stress) and area (t_(6)_ = 3.172, ∗*p* < 0.05, stress vs control in adolescence, t_(6)_ = 1.516, *p* = 0.1802, adolescence vs adulthood in stress) were significantly lower in the stress group than in the control group during adolescence (Fig. 4K and L). To further examine this phenomenon, we analyzed the expression of Egr2 (Supplementary Fig. 3A–F). Immunofluorescence staining revealed markedly increased Egr2 levels in both the hippocampus and hypothalamus of the stress group in adolescence compared with those in the stress group in adulthood (Supplementary Figs. 3B, C, E, F). Among these three proteins, the protein expression patterns of Fabp7 and CxCl12 in hippocampus were identical to their mRNA expression patterns, but Egr2 was not.

These results suggest that the stress model used in this study significantly modulates the protein expression of Fabp7 and Cxcl12 in both the hippocampus and hypothalamus in an age-dependent manner. Specifically, Fabp7 expression was upregulated following adolescence stress, whereas Cxcl12 expression was downregulated during the same developmental stage. In contrast, both proteins presented opposite expression patterns under adulthood stress conditions. These results suggest that the opposite effects of chronic stress across age groups may be attributed to biologically relevant molecular changes.

## 4 Discussion

This study examined how the timing of stress exposure during either adolescence or adulthood modulates behavioral and molecular adaptations in mice subjected to PTSD-like stress combined with social isolation. Behavioral assessments revealed a clear age-dependent dichotomy: Adolescence stress was associated with increased social dominance, whereas early adulthood and adulthood stress led to a reduction in dominant behavior. These opposing behavioral outcomes demonstrate that the developmental stage significantly influences the direction and impact of stress-induced behavioral changes. Interestingly, in the early adulthood stress group, there was no change in immobility time in the TST unlike the adolescence and adult stress groups. This difference in behavioral changes may have contributed to the lack of clear separation between the control and stress groups in the PCA plot of early adulthood. In future follow-up studies, examining DEG changes in the early adulthood group to determine the association between gene expression changes and behavioral changes may provide meaningful insights.

Recent studies have shown that neurogenesis and anxiety during adolescence predict and regulate social hierarchy in adult mice (Grieco et al., 2025). Specifically, individuals with lower neurogenesis and higher anxiety during adolescence were more likely to become dominant when forming new groups (Grieco et al., 2025). Our results revealed that the *Cxcl12* gene, which is involved in neurogenesis, neuron differentiation, and neuron development, was specifically downregulated in the adolescence stress group, and its protein expression was also reduced. Although we did not examine neurogenesis-related factors such as BrdU and anxiety scores, in line with the study by Grieco et al., the reason why the stress-induced increase in dominant behavior was observed only in the adolescent stress group in this study may be related to the decrease in neurogenesis-related genes.

Transcriptome analysis of the hippocampus revealed unique gene expression signatures associated with the stress response across each life stage. Hierarchical clustering identified two major gene modules. A 75-gene module, including *Fabp7*, *Egr2*, and *B3gnt*, is upregulated in adolescence and downregulated in adulthood in response to stress. A 165-gene module, including *Cxcl12*, *Stra6*, and *Zfhx3*, is downregulated during adolescence and upregulated during adulthood in response to stress. Gene ontology enrichment analysis subdivided these gene modules into subsets of genes involved in neural functions, such as neurogenesis, neuronal differentiation, neuronal development, brain development, and learning and memory. Thirteen upregulated and 22 downregulated genes were classified as genes involved in neural functions, and among them, *Fabp7* and *Cxcl12* were confirmed to be consistent with the results of transcriptome analysis, mRNA expression, and protein expression in the hippocampus through qPCR and immunofluorescence staining.

Fabp7 is robustly expressed in radial glia, astrocytes, and neural stem/progenitor cells, where it plays a pivotal role in regulating lipid metabolism and neuronal differentiation (Boneva et al., 2011; Hamilton et al., 2024; Pose-Méndez et al., 2024). Functioning as a fatty acid chaperone, Fabp7 facilitates the intracellular transport of polyunsaturated fatty acids such as DHA, influencing neuroplasticity via gene transcription and PUFA-GPR40 signaling (Boneva et al., 2011; Ebrahimi et al., 2016; Lenz et al., 2023; Oeemig et al., 2009). Notably, upon PUFA binding, Fabp7 translocates to the nucleus, where it modulates histone acetylation and gene expression, which are critical for neural progenitor proliferation and differentiation (Liu et al., 2010; Matsumata et al., 2012).

Furthermore, Fabp7 is upregulated in response to early-life stress and cerebral ischemia, especially in hippocampal regions, suggesting an adaptive mechanism promoting synaptic remodeling, neurogenesis, and dendritic complexity (Kato et al., 2020; Liu et al., 2016; Ma et al., 2010). This finding is supported by evidence that Fabp7 deficiency impairs astrocyte-mediated lipid signaling, reduces dendritic spine density, and disrupts excitatory synapse formation. In addition, through its interaction with caveolin-1, Fabp7 modulates lipid raft dynamics and neurotrophic factor signaling, potentially contributing to stress adaptation and HPA axis regulation (Chistiakova & Savost'ianov, 2011; Geng et al., 2025; Kagawa et al., 2015).

Cxcl12, also known as stromal cell-derived factor-1, plays a critical role in the CNS by orchestrating key neurodevelopmental and neuroadaptive processes (Gronthos and Zannettino, 2007; Zhu et al., 2017). During brain development, it guides the migration and spatial positioning of cortical interneurons and dopaminergic neurons, particularly within the ventral tegmental area and substantia nigra (Nishikawa et al., 2003; Yamagishi et al., 2020). In the mature brain, Cxcl12 enhances synaptogenesis by promoting axonal elongation, dendrite formation, and synaptic connectivity, thereby facilitating circuit integration and synaptic remodeling (Li et al., 2021; Nash and Meucci, 2014). It also regulates dendritic spine density and modulates glutamatergic signaling through NMDA receptor activity and glutamate release, mechanisms essential for maintaining excitatory neurotransmission and plasticity (Kessels et al., 2013; Nicolai et al., 2010; Ultanir et al., 2007).

In addition to its structural and synaptic functions, Cxcl12 confers neuroprotection under excitotoxic conditions by modulating intracellular pathways such as retinoblastoma protein phosphorylation and histone deacetylation (Khan et al., 2008, 2011). Additionally, Cxcl12 supports neural progenitor survival and adult neurogenesis through Cxcl7-mediated endocytosis signaling (Wang et al., 2016; Zhu et al., 2012). It also facilitates glutamatergic transmission in the basolateral amygdala, contributing to anxiety regulation under inflammatory stress (Karst and Joëls, 2016; Zhong et al., 2022). Collectively, these functions position Cxcl12 as a central regulator of neuronal migration, survival, synaptic integration, and behavior across developmental and stress-related contexts.

Consistent with these findings, our study revealed significant downregulation of Cxcl12 in the hippocampus and hypothalamus of mice exposed to adolescence stress. This reduction may disrupt neurogenesis and network assembly, compromise neurotransmitter regulation, and weaken resilience mechanisms. These results suggest that Cxcl12 might be modulated to maintain stress-adaptive neuronal function.

In the hypothalamus, Fabp7 contributes to energy homeostasis, neuroendocrine stress responses, and circadian gene regulation (Gerstner et al., 2008; Hamilton et al., 2022; Lu et al., 2025; Yasumoto et al., 2018). Its role in modulating leptin sensitivity, endocannabinoid signaling, and astrocytic metabolic function indicates a broad regulatory influence on hypothalamic output relevant to behavioral adaptation (Geng et al., 2025; Hamilton et al., 2022; Lu et al., 2025; Yasumoto et al., 2018). Conversely, Cxcl12 regulates vasopressin release, neurogenesis, and immune responses in the hypothalamus, and its downregulation has been linked to altered feeding behavior (Barbieri et al., 2014; Petković et al., 2013; Stumm et al., 2002; Trecki and Unterwald, 2009).

The inverse expression dynamics of Fabp7 and Cxcl12 suggest an intricate regulatory balance between resilience-promoting and vulnerability-associated molecular cascades in response to stress. The upregulation of Fabp7 may support neuroadaptive capacity, whereas the suppression of Cxcl12 may reflect heightened sensitivity to stress-induced neural destabilization (Li et al., 2019; Muneer et al., 2017; Straiker et al., 2023). Both proteins indirectly interact with canonical pathways, including the ERK, Notch, MAPK, and PPARγ pathways, and may affect key transcriptional regulators, such as NeuroD1 and Egr1 (Baek et al., 2003; Cheng et al., 2017; Iyoda et al., 2012; Lee et al., 2020; Sabol et al., 2024; Slipicevic et al., 2008; Sun et al., 2022; Zhang et al., 2024). These studies suggest that Fabp7 and Cxcl12 in the hippocampus and hypothalamus might perform circuit-level interactions to modulate neuronal function and behavioral outcomes.

These molecular alterations may indicate the bidirectional changes in social dominance behavior observed in our chronic stress model. Upregulated Fabp7 may support adaptive responses via enhanced astrocyte‒neuron metabolic coupling and stress buffering (Bresque et al., 2025; Chen et al., 2025; Hamilton et al., 2024), whereas Cxcl12 suppression could impair neuroendocrine stability (Barbieri et al., 2014). These contrasting transcriptional patterns observed in the hypothalamus and hippocampus support a converging model in which stress-induced gene regulation via interconnected limbic circuits contributes to the emergence and persistence of long-term behavioral phenotypes.

Comparative analysis with previous transcriptome datasets from PTSD models revealed partial differences in gene expression profiles (Pan et al., 2021; Stankiewicz et al., 2022). For example, *Fabp7* has been reported to be downregulated in a PTSD-like mouse model via a single prolonged stress or footshock protocol (Stankiewicz et al., 2022), which differs from our findings that *Fabp7* was upregulated in our chronic stress model. Similarly, *Cxcl12* has been suggested as a gene whose expression is altered in response to trauma-related stress (Pan et al., 2021). For example, Cxcl12 was identified as a potential biomarker for trauma and/or PTSD, with a tendency toward increased expression in PTSD patients compared with controls, although the difference was not statistically significant (Pan et al., 2021). In contrast, we observed significant downregulation of *Cxcl12* in our dual-stressor paradigm, in which PTSD was combined with social isolation. Furthermore, comparative analysis with the GO-term integrated transcriptome database revealed that several genes prioritized in our study, such as *Arc*, *Cxcl12*, *Prox1*, *Bmp6*, *Lef1*, *Tgfbr2*, and *Rac3*, were also highly ranked in previous PTSD-related studies (Stankiewicz et al., 2022). Interestingly, *Fabp7* and *Cxcl12* have also been reported at high frequencies, but their directionality in our study differed from that observed in previous models. This discrepancy may be explained by differences in the animal's developmental stage (Adolescence versus Adulthood), the nature of the stressors (combined versus single traumatic factors), or compensatory mechanisms that operate under chronic social stress. These diverse conditions suggest that they may act as contextual factors shaping the diversity of gene expression in stress responses.

Transcriptome and qPCR analyses of the *Egr2*, *B3gnt2*, *Stra6*, and *Zfhnx3* genes were consistent in the hippocampus, but the mRNA expression patterns of *B3gnt2*, *Stra6*, and *Zfhnx3* genes in the hypothalamus differed from those in the hippocampus, and Egr2 protein expression did not match its mRNA expression in the hippocampus. However, *Egr2* is known to be an important transcription factor in regulating nervous system development and nervous system functions such as memory and learning, and is associated with neurobehavioral abnormalities, memory, and ataxia (Le et al., 2005; McCowan et al., 2021; Poirie et al., 2007). In addition, Zfhnx3 is known to play an important role in circadian rhythm, neural differentiation, sleep, and nervous system development (Wilcox et al., 2017, 2021). Since all four genes are neural function-related genes that showed significant changes in the adolescent stress group, they are likely to affect direct and indirect stress-related changes, and further studies are needed in the future.

While our findings provide valuable insights, several methodological limitations should be acknowledged. Because only male mice were used, conclusions regarding sex-specific stress responses are limited. Whole-cell RNA extraction methods present challenges in identifying cell-type-specific transcriptome changes, which could be addressed through future single-cell or spatial transcriptome analyses. Furthermore, the lack of direct comparative analysis with PTSD-only or social isolation-only models limits the ability to analyze the contributions of individuals and interstressors. Addressing these gaps in future research will further enhance the interpretive depth and potential of the results of this study.

## 5 Conclusions

Our findings highlight the critical role of developmental timing in stress-induced molecular and behavioral changes. Mice exposed to stress during adolescence and adulthood presented similar levels of depressive-like behavior but differed in gene expression patterns and social dominance patterns. These results suggest that stress exerts differential effects on gene and protein expression at different developmental stages, potentially leading to conflicting behavioral outcomes. Specifically, these findings raise important questions about how molecular changes, such as the age-dependent upregulation of *Fabp7* and downregulation of *Cxcl12* in adolescence because of stress, contribute to physiological changes in the brain, including changes in neural circuitry, neurodevelopment, and synaptic function. Further studies are needed to determine how these gene-specific changes play a role in regulating social dominance and other complex behaviors following stress exposure in an age-dependent manner.

## CRediT authorship contribution statement

**Moonseok Choi:** Data curation, Formal analysis, Writing – original draft. **Jisu Jeong:** Formal analysis, Writing – original draft. **Dongsoo Kim:** Conceptualization, Data curation, Formal analysis, Writing – original draft. **Hong Seok Choi:** Formal analysis, Writing – review & editing. **Junghwa Ryu:** Writing – review & editing. **Hye Jin Choi:** Writing – review & editing. **Mookyung Cheon:** Conceptualization, Writing – review & editing. **Yun Ha Jeong:** Conceptualization, Funding acquisition, Supervision, Writing – original draft, Writing – review & editing.

## Funding

This work/research was supported by the Korea Brain Research Institute basic research program funded by the Ministry of Science, ICT (MSIT) (25-BR-02-04, 25-BR-04-01); and by the National Research Foundation grants funded by the Korean government (MSIT) (NRF-2019R1C1C1011390).

## Declaration of competing interest

The authors declare that they have no conflict of interest.

## Data Availability

Data will be made available on request.

## Glossary

(AS): acute stress
(ANOVA): analysis of variance
(ARRIVE): Animal Research: Reporting of In Vivo Experiments
(BP): biological process
(CORT): corticosterone
(DEGs): differentially expressed genes
(ELISA): enzyme-linked immunosorbent assay
(FST): forced swimming test
(GO): Gene Ontology
(HSD): honestly significant difference
(KEGG): Kyoto Encyclopedia of Genes and Genomes
(NB): negative binomial
(OCT): optimal cutting temperature
(OCT): optimal cutting temperature
(PTSD): posttraumatic stress disorder
(qPCR): Quantitative PCR
(QL): quasilikelihood
(ROI): regions of interest
(SPS): single-prolonged stress
(SI): social isolation
(TST): tail suspension test
(TMM): trimmed mean of M-values
