# Supplementary material for: Age-dependent effects of chronic traumatic and social isolation stress on mice social behavior
Source: Neurobiol Stress. 2025 Nov 27;40:100773. doi: 10.1016/j.ynstr.2025.100773 (PMC12721305; doi:10.1016/j.ynstr.2025.100773)
Supplement: Multimedia component 1 [file mmc1.docx]

**Age-dependent effects of chronic traumatic and social isolation stress on mice social behavior**

**Supplemental Material**


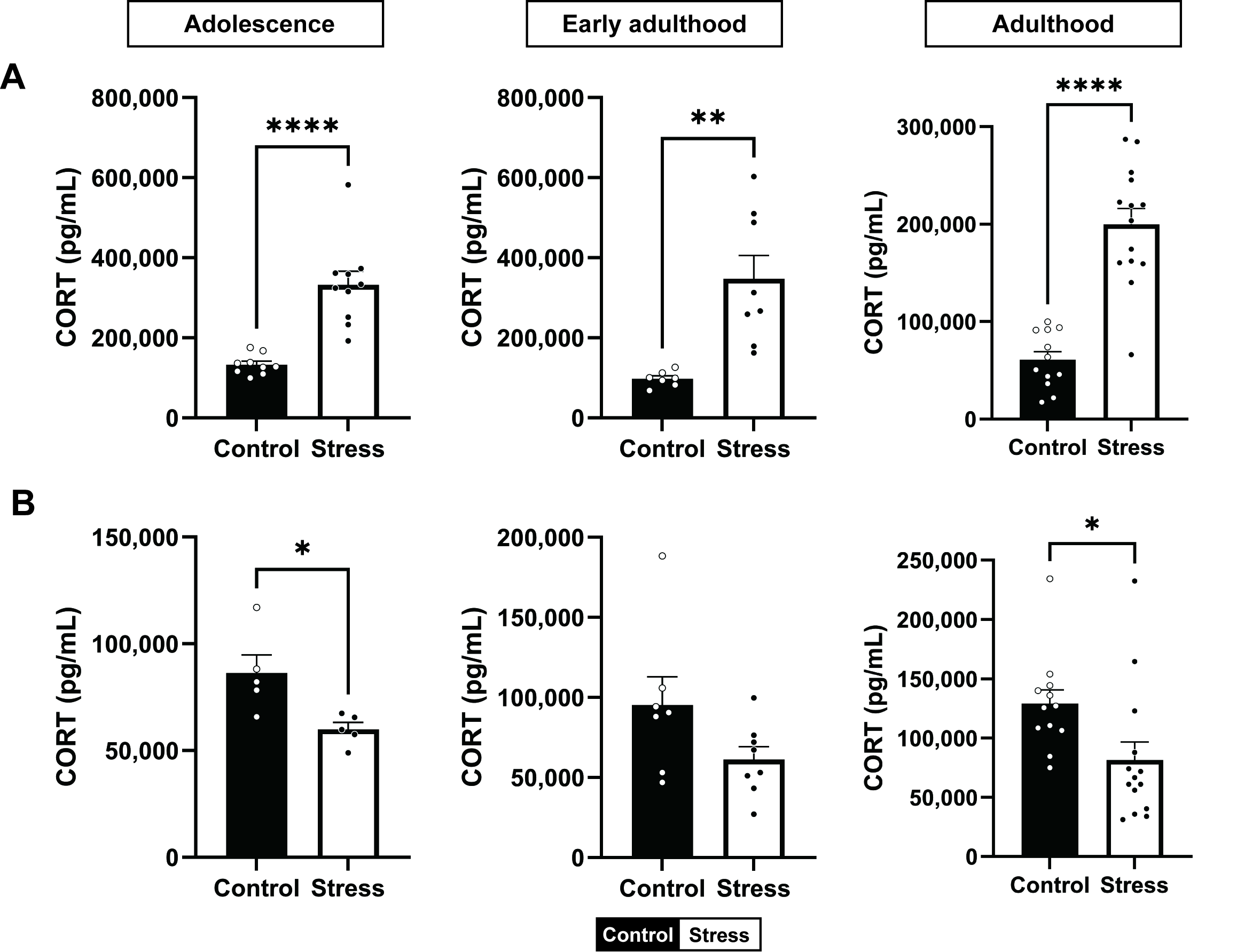


**Supplemental Fig. 1. Effects of chronic traumatic and chronic social isolation (SI) stress on serum corticosterone (CORT) levels across life cycle.** (A) Effects of SPS on serum CORT level (Student’s *t*-test; adolescence, n = 9–10 mice per group, *****p* < 0.0001; early adulthood, n = 7–8 mice per group, ***p* < 0.01; adulthood, n = 12–14 mice per group, *****p* < 0.0001). (B) Effects of SI on serum CORT level (Student’s *t*-test; adolescence, n = 5 mice per group, **p* < 0.05; early adulthood, n = 7–8 mice per group, ns; adulthood, n = 12–14 mice per group, **p* < 0.05). Results are presented as the mean ± SEM. Statistical analysis was performed using Student’s *t*-test (unpaired). **p* < 0.05, ***p* < 0.01 *****p* < 0.0001.


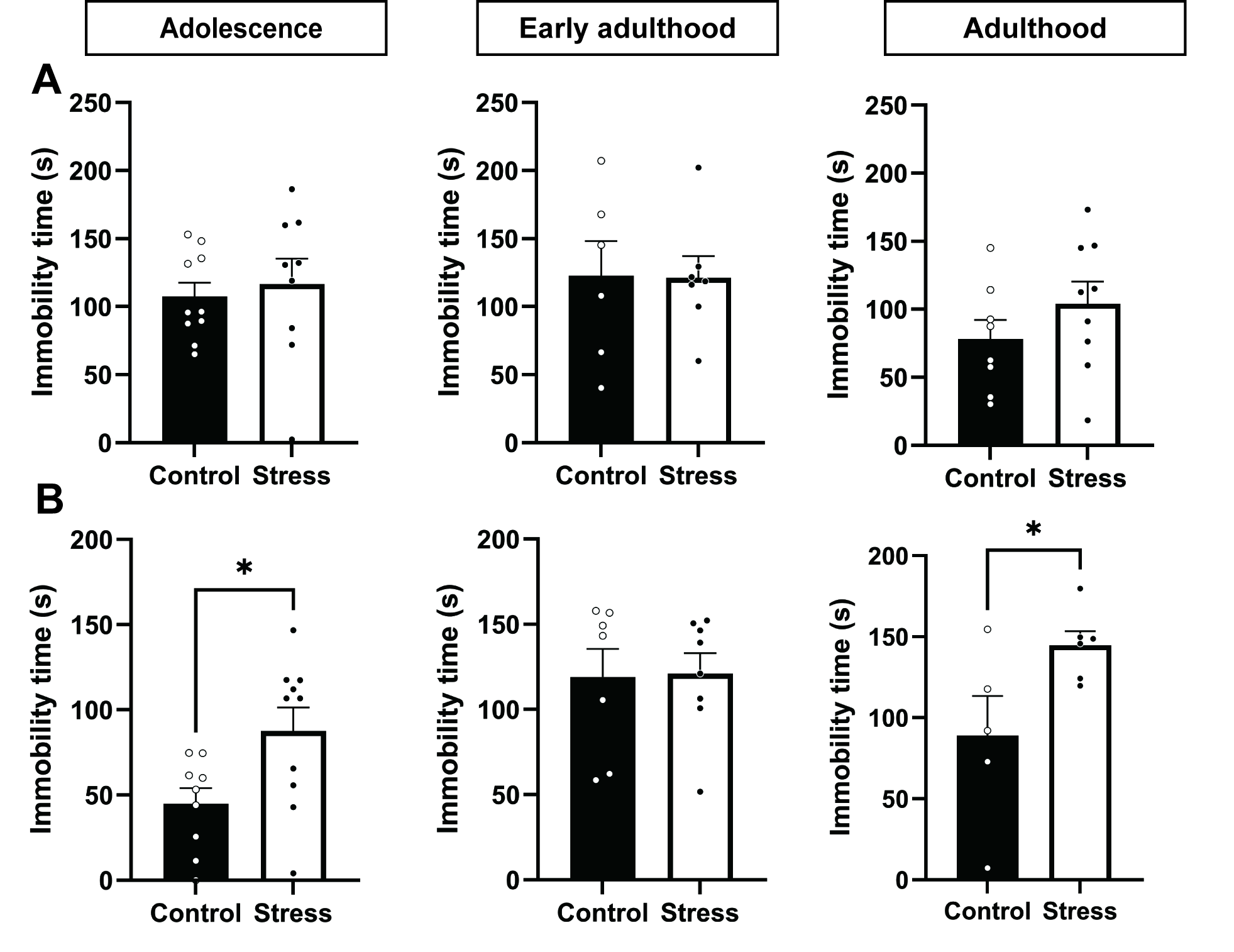


**Supplemental Fig. 2. Validation of chronic traumatic and chronic social isolation stress across life cycle using behavioral analysis.** (A) Bar graph showing immobility time during the forced swim test (FST) (Student’s *t*-test; adolescence, n = 9–10 mice per group, ns; early adulthood, n = 6–7 mice per group, ns; adulthood, n = 8–9 mice per group, ns). (B) Bar graph showing immobility time during the tail suspension test (TST) (Student’s *t*-test; adolescence, n = 9–10 mice per group, **p* < 0.05; early adulthood, n = 7–8 mice per group, ns; adulthood, n = 5–6 mice per group, **p* < 0.05). Results are presented as the mean ± SEM. Statistical analysis was performed using Student’s *t*-test (unpaired). **p* < 0.05.

**Supplemental Table. 1**

***P*-values and log_2_FC changes for 35 genes in Adolescence: Stress vs Control comparison**

| **Gene symbols** | **Gene name** | **Gene ID** | **Entrez ID** | **Adolescence** | |
| --- | --- | --- | --- | --- | --- |
|  |  |  |  | **(Stress vs Control)** | |
|  |  |  |  | ***P*-value** | **Log_2_FC** |
| **Upregulated Genes** | | | | | |
| *Egr2* | Early Growth Response 2 | ENSMUSG  00000037868 | 13654 | 0.0007 | 0.897 |
| *Arc* | Activity Regulated Cytoskeleton Associated Protein | ENSMUSG  00000022602 | 11838 | 2.36E-06 | 0.866 |
| *Dnmt3b* | DNA Methyltransferase 3 Beta | ENSMUSG  00000027478 | 13436 | 0.0328 | 0.772 |
| *B3gnt2* | UDP-GlcNAc:BetaGal Beta-1,3-N-Acetylglucosaminyltransferase 2 | ENSMUSG  00000051650 | 53625 | 0.0241 | 0.727 |
| *Col6a1* | Collagen Type VI Alpha 1 Chain | ENSMUSG  00000001119 | 12833 | 0.0040 | 0.694 |
| *Fabp7* | Fatty acid binding protein 7 | ENSMUSG  00000019874 | 12140 | 0.0009 | 0.625 |
| *Rnd1* | Rho Family GTPase 1 | ENSMUSG  00000054855 | 223881 | 0.0021 | 0.617 |
| *Grp* | Gastrin Releasing Peptide | ENSMUSG  00000024517 | 225642 | 0.0020 | 0.509 |
| *Nr2f6* | Nuclear Receptor Subfamily 2 Group F Member 6 | ENSMUSG  00000002393 | 13864 | 0.0080 | 0.499 |
| *Ndp* | Norrin Cystine Knot Growth Factor NDP | ENSMUSG  00000040138 | 17986 | 0.0328 | 0.470 |
| *Hes1* | Hes Family BHLH Transcription Factor 1 | ENSMUSG  00000022528 | 15205 | 0.0050 | 0.450 |
| *Rac3* | Rac Family Small GTPase 3 | ENSMUSG  00000018012 | 170758 | 0.0146 | 0.424 |
| *Lsm1* | LSM1 Homolog, MRNA Degradation Associated | ENSMUSG  00000037296 | 67207 | 0.0071 | 0.423 |
| **Downregulated Genes** | | | | | |
| *Gh* | Growth hormone | ENSMUSG  00000020713 | 145999 | 0.030823 | -3.343 |
| *Mc3r* | Melanocortin 3 Receptor | ENSMUSG  00000038537 | 17201 | 2.53E-05 | -2.941 |
| *Ccl3* | C-C Motif Chemokine Ligand 3 | ENSMUSG  00000000982 | 20302 | 0.0013 | -2.318 |
| *Avp* | Arginine Vasopressin | ENSMUSG  00000037727 | 11998 | 0.0127 | -1.643 |
| *Chrnb4* | Cholinergic Receptor Nicotinic Beta 4 Subunit | ENSMUSG  00000035200 | 108015 | 0.0012 | -1.629 |
| *S1pr4* | Sphingosine-1-Phosphate Receptor 4 | ENSMUSG  00000044199 | 13611 | 0.0473 | -1.368 |
| *Cdkn1c* | Cyclin Dependent Kinase Inhibitor 1C | ENSMUSG  00000037664 | 12577 | 0.0009 | -1.074 |
| *Bmp6* | Bone Morphogenetic Protein 6 | ENSMUSG  00000039004 | 12161 | 0.0004 | -1.032 |
| *Lcn2* | Lipocalin 2 | ENSMUSG  00000026822 | 16819 | 0.0494 | -0.919 |
| *Bmp7* | Bone Morphogenetic Protein 7 | ENSMUSG  00000008999 | 12162 | 3.83E-05 | -0.810 |
| *Zfhx3* | Zinc Finger Homeobox 3 | ENSMUSG  00000038872 | 11906 | 0.0102 | -0.714 |
| *Lef1* | Lymphoid Enhancer Binding Factor 1 | ENSMUSG  00000027985 | 16842 | 0.0023 | -0.655 |
| *Sema3g* | Semaphorin 3G | ENSMUSG  00000021904 | 218877 | 0.0025 | -0.648 |
| *Tgfbr2* | Transforming Growth Factor Beta Receptor 2 | ENSMUSG  00000032440 | 21813 | 5.13E-05 | -0.614 |
| *Stra6* | Signaling Receptor And Transporter Of Retinol STRA6 | ENSMUSG  00000032327 | 20897 | 0.0001 | -0.594 |
| *Gata2* | GATA Binding Protein 2 | ENSMUSG  00000015053 | 14461 | 0.0106 | -0.564 |
| *Lamc3* | Laminin Subunit Gamma 3 | ENSMUSG  00000026840 | 23928 | 0.0107 | -0.541 |
| *C3ar1* | Complement C3a Receptor 1 | ENSMUSG  00000040552 | 12267 | 0.0184 | -0.528 |
| *Cxcl12* | C-X-C Motif Chemokine Ligand 12 | ENSMUSG  00000061353 | 20315 | 0.00004 | -0.499 |
| *Tnfrsf1b* | TNF Receptor Superfamily Member 1B | ENSMUSG  00000028599 | 21938 | 0.0349 | -0.477 |
| *Prox1* | Prospero Homeobox 1 | ENSMUSG  00000010175 | 19130 | 0.0283 | -0.454 |
| *Clic5* | Chloride Intracellular Channel 5 | ENSMUSG  00000023959 | 224796 | 0.0230 | -0.428 |

**Supplemental Table 2**

**List of primer sequences for qRT-PCR**

| **Gene symbols** | **Primer** | **Sequence (5'->3')** |
| --- | --- | --- |
| *Gapdh* | Forward | AGGTCGGTGTGAACGGATTTG |
|  | Reverse | TGTAGACCATGTAGTTGAGGTCA |
| *Egr2* | Forward | TCAGTGGTTTTATGCACCAGC |
|  | Reverse | GAAGCTACTCGGATACGGGAG |
| *Fabp7* | Forward | AAGTGGGAAACGTGACCAAAC |
|  | Reverse | CAACCGAACCACAGACTTACAG |
| *B3gnt2* | Forward | ATACTGGAACAGGGAACAGGAG |
|  | Reverse | GTCTGGTTCACAATAGCTCAGG |
| *Cxcl12* | Forward | TGCATCAGTGACGGTAAACCA |
|  | Reverse | TTCTTCAGCCGTGCAACAATC |
| *Stra6* | Forward | ACAGAGCTAAGCAGACAGGAGG |
|  | Reverse | GTGAGGCCGCAGTAGTTGT |
| *Zfhx3* | Forward | GGGGAGATCGTCTACCAGC |
|  | Reverse | TCAAACCATTTCCCGAAGGATG |

**Supplemental Fig. 3. Effects of chronic stress on the protein expression levels of Egr2 in the hippocampus and hypothalamus.** (A) Representative confocal images showing the immunofluorescence staining of Egr2 (green) in the hippocampus of the control and stress groups. Nuclei were counterstained with DAPI (blue). Scale bar: 100 μm, Zoom: 10 μm. (B, C) Quantification of the intensity (adolescence vs adulthood in stress group, n = 4–5 mice per group, ****p* < 0.001) and area (adolescence vs adulthood in stress group, n = 3–5 mice per group, *****p* < 0.0001) of Egr2 immunofluorescence in the hippocampus of control and stress mice. (D) Representative confocal images showing the immunofluorescence staining of Egr2 (green) in the hypothalamus of the control and stress groups. Scale bar: 100 μm, Zoom: 10 μm. (E, F) Quantification of the intensity (adolescence vs adulthood in stress group, n = 3–5 mice per group, ***p* < 0.01) and area (adolescence vs adulthood in stress group, n = 4–5 mice per group, **p* < 0.05) of Egr2 immunofluorescence in the hypothalamus of control and stress groups. All data are presented as mean ± SEM. **p* < 0.05, ***p* < 0.01, ****p* < 0.001, *****p* < 0.0001 stress vs control in the adolescence or adolescence vs adulthood in the stress group (unpaired *t*-test).
